# Henry County Medical Society

**Published:** 1856-04

**Authors:** E. Pomeroy, T. D. Fitch

**Affiliations:** President; Secretary


*Henry County Medical Society.*

Pursuant to previous notice, the physicians of Henry County met at Cambridge, March 3, 1856, to organize a County Medical Society.

On motion, E. Pomeroy, M.D., of Geneseo, was called to the chair, and T. D. Fitch, of Kewanee, chosen Secretary.

On motion, we proceeded to the organization of a Medical Society.

On motion, a committee of three, consisting of Drs. O. H. Edwards, T. D. Fitch, and R. J. Stough, were appointed to present a Constitution and By-Laws for said Society.

The committee submitted the following Constitution and By-Laws, which, on motion, were approved and adopted article by article:—

CONSTITUTION.

Art. I. This Society shall be known and designated the *Henry County Medical Society.*

Art. II. The physicians present at the adoption of this Constitution shall be members of the Society.

Art. III. Any regular graduate of an orthodox school, or any physician passing a satisfactory examination before the Board of Censors, may become a member by a vote of the Society.

Art. IV. The officers of the Society shall consist of a President, Vice-President, Secretary, who shall act as Treasurer, and three Censors, to be elected annually by a majority vote of all the members present.

Art. V. The Society shall be governed by the code of ethics adopted by the Illinois State Medical Society.

Art. VI. This Constitution may be altered or amended by a vote of two-thirds of the members present at any regular meeting.

BY-LAWS.

Art. I. Each member shall report a case or read an Essay at every regular meeting, and an additional Essay shall be read at each meeting—the Essayist to be appointed at the previous meeting, in alphabetical order, excepting the annual address, which shall be by appointment.

Art. II. The report of cases and essays made before the Society at its regular meetings, shall become the property of the Society.

Art. III. Any member who has conducted himself properly during his membership, shall, if he require, receive a certificate of character signed by the President and Secretary.

Art. IV. Every member failing to comply with the provisions of Art. 1st of By-Laws, shall for every offence be fined one dollar.

On motion, the Society proceeded to the election of officers, when the following officers were duly elected:—

Dr. E. POMEROY, *President.*

Dr. J. M. WINN, *Vice-President.*

Dr. T. D. FITCH, *Secretary and Treasurer.*

Drs. H. Luddington, R. J. Stough, and O. H. Edwards, *Censors.*

On motion of Dr. Luddington, Dr. T. D. Fitch was appointed a delegate to the American Medical Association.

Horace Luddington, M.D., of Geneseo, was appointed to deliver the annual address before the Society in June.

Dr. Stough moved that the Secretary be instructed to forward the proceedings of this meeting to the *North-Western Medical and Surgical Journal*, and our county papers for publication—carried.

On motion of Dr. Fitch, Society adjourned to meet in Keewanee on the first Monday in June next.

E. POMEROY, M.D., *President.*

T. D. FITCH, M.D., *Secretary.*

